# R-Loops in Genome Instability and Cancer

**DOI:** 10.3390/cancers15204986

**Published:** 2023-10-14

**Authors:** Fang Li, Alyan Zafar, Liang Luo, Ariana Maria Denning, Jun Gu, Ansley Bennett, Fenghua Yuan, Yanbin Zhang

**Affiliations:** 1 Department of Biochemistry & Molecular Biology, Miller School of Medicine, University of Miami, Miami, FL 33136, USA; 2 Department of Molecular and Cellular Pharmacology, Miller School of Medicine, University of Miami, Miami, FL 33136, USA

**Keywords:** R-loops, RNA–DNA hybrid, genome instability, double-strand breaks, transcription–replication conflicts, DNA repair, transcription-coupled homologous recombination, cancer

## Abstract

**Simple Summary:**

R-loops are three-stranded structures consisting of an RNA–DNA hybrid and an unpaired single-stranded DNA (ssDNA), with biological implications in cellular physiology and pathological conditions. R-loops interfere with DNA repair pathways and activate oncogenes, leading to dysregulated cell proliferation, genome instability, and cancer development. R-loops accumulate in several types of cancer cells, including breast, ovarian, prostate, and lung cancer. Studying the biological roles of R-loops in cancer development is potentially beneficial for innovative diagnostic and treatment approaches for cancer. In this review, we focus on recent advances in R-loops’ roles in genome instability, DNA repair, and oncogenic events.

**Abstract:**

R-loops are unique, three-stranded nucleic acid structures that primarily form when an RNA molecule displaces one DNA strand and anneals to the complementary DNA strand in a double-stranded DNA molecule. R-loop formation can occur during natural processes, such as transcription, in which the nascent RNA molecule remains hybridized with the template DNA strand, while the non-template DNA strand is displaced. However, R-loops can also arise due to many non-natural processes, including DNA damage, dysregulation of RNA degradation pathways, and defects in RNA processing. Despite their prevalence throughout the whole genome, R-loops are predominantly found in actively transcribed gene regions, enabling R-loops to serve seemingly controversial roles. On one hand, the pathological accumulation of R-loops contributes to genome instability, a hallmark of cancer development that plays a role in tumorigenesis, cancer progression, and therapeutic resistance. On the other hand, R-loops play critical roles in regulating essential processes, such as gene expression, chromatin organization, class-switch recombination, mitochondrial DNA replication, and DNA repair. In this review, we summarize discoveries related to the formation, suppression, and removal of R-loops and their influence on genome instability, DNA repair, and oncogenic events. We have also discussed therapeutical opportunities by targeting pathological R-loops.

## 1. Introduction

R-loops are three-stranded structures comprising RNA–DNA hybrids and ssDNA displaced from the non-template strand [1,2]. Once formed, R-loops are typically thermodynamically stable due to the stabilizing interaction between RNA and DNA [3]. R-loops were initially visualized in 1976 through electron microscopy when they were formed in vitro in the presence of 70% formamide. Interestingly, R-loops remained intact following the removal of formamide [4]. Decades of research have revealed different key biological roles of R-loops in multiple organisms, including chromatin organization and chromosome segregation [5,6,7]. The prevalent model of biological R-loop formation exhibits the newly synthesized RNA strand competing with the non-template DNA strand for re-annealing to the template DNA strand by leaving the RNA exit channel of the RNA polymerase complex as it travels along the DNA template [8].

Recent advancements in genome-wide mapping methods reveal that R-loops are abundant in the mammalian genome, predominantly in GC-rich regions at the 5′ promoter and 3′ end regions of many genes [9,10,11]. While R-loops are notably known for their detrimental effects on DNA stability and gene expression, growing evidence suggests that R-loops may play positive roles in essential biological processes. In gene regulation, R-loops play a multifaceted role by acting as gene promoters, aiding in transcriptional termination, facilitating centromere function, promoting DNA methylation, and benefiting histone modification [10,12,13]. Moreover, R-loops play a beneficial role in maintaining genome stability by promoting DNA double-strand break (DSB) repair, preserving telomere integrity, conserving DNA topology by acting as “stress relief valves”, and aiding in DNA replication and immunoglobulin class-switch recombination [14]. Conversely, R-loops also contribute to aberrant gene regulation, genome instability, and DNA damage by serving as primers for abnormal replication, acting as effectors of transcription stress, and promoting transcription–replication collision. Thus, R-loops can contribute to DNA damage, disrupt genome integrity, promote activation of DNA damage response, and halt RNA processing [2,12].

This review comprehensively revisits and focuses on recent advancements in R-loops’ biological effects under physiological and pathological conditions. Additionally, we summarize factors and pathways related to R-loops formation, stabilization, and resolution in different cellular processes and examine their influences on DNA replication, DSB repair, and chromatin structure. Finally, we discuss possible causes of altered R-loop metabolism on cancer development and potential opportunities for targeting unscheduled R-loops in cancer therapy.

## 2. R-Loops’ Formation in Physiological and Pathological Conditions

R-loops form naturally in different cellular, chromosomal, and gene contexts, including replication, transcription, and DNA repair. They usually accumulate in specific genome regions, including telomeres, centromeres, and mitochondrial DNA (mtDNA) [5]. R-loops are dynamic structures that are prevalent in the whole genome, accounting for up to 5% of the mammalian genome and present on thousands of copies of mtDNA per cell [15,16,17].

### 2.1. R-Loops during Transcription

During transcription, the nascent RNA transiently anneals back onto the DNA template within the active site of the RNA polymerase, forming a short, transient RNA–DNA hybrid called an R-loop [18]. R-loops play a critical role in the transcription process and regulate gene expression in several ways, such as by influencing promoter directionality, facilitating transcriptional termination, and participating in class-switch recombination in immunoglobulin-producing cells [19].

R-loop accumulation has been observed to interfere with the transcriptional process carried out by RNA polymerase I (Pol I), particularly in the 5′ regions of 18S genes [20]. RNA Pol I is responsible for more than 60% of total cellular transcription [21]; thus, proper transcriptional control by RNA Pol I is critical to maintaining genomic stability. When R-loops form during transcription, they create a physical blockade to the progression of the RNA Pol I machinery, leading to the stalling of the transcriptional process and the subsequent onset of genomic instability [22]. The presence of these R-loops can cause the replication machinery to stall [23], accelerating replication stress, DNA breaks, and other forms of genomic instability [24]. This sequence of events could consequently activate cell mechanisms linked to DNA damage responses and repair, induce a halt in the cell cycle, and in specific instances, trigger controlled cell death by apoptosis [25]. It is noteworthy to add that ribosomal DNA (rDNA) genes are only located in the nucleolar organizer regions at the short arms of the five acrocentric chromosomes [26]; thus, genomic instability originated from the stalling of RNA Pol I may not be seen in the whole genome. Moreover, the precise biochemical processes by which R-loop accumulation impedes RNA Pol I transcription remain unclear. Understanding these biochemical mechanisms might bear significance in comprehending disorders in which rRNA transcription is unregulated, like cancer, a condition marked by heightened protein synthesis and cell proliferation [2].

During the gene transcription by RNA polymerase II (Pol II), which is primarily responsible for transcribing mRNA and several non-coding RNA molecules, R-loops typically form co-transcriptionally promoter sites, as well as transcription termination sites [27,28]. Typically, an R-loop forms when the newly synthesized RNA molecule hybridizes with the DNA template strand, leaving the non-template strand unpaired, creating a physical barrier for RNA Pol II and stalling its progression along the DNA strand [27]. Stalling of RNA Pol II can produce truncated transcripts, which may be non-functional and result in aberrant proteins if translated [29]. Moreover, RNA Pol II stalling can lead to collisions with the DNA replication machinery, promoting replication stress and potentially DNA double-strand breaks, a significant source of genomic instability [30,31]. It is also important to note that chronic R-loop accumulation can affect the transcription of nearby genes, altering the chromatin landscape and causing global changes in gene expression. Subsequent alterations in the chromatin structure, overall gene expression, and potential DNA damage are implicated in various diseases, including neurodegenerative and oncogenic disorders [2,5,13].

Under normal growth conditions, the 5S rRNA genes exhibit a propensity for R-loop formation [32,33], unlike other RNA Pol III genes (tRNA genes), which only demonstrate R-loop accumulation without ribonucleases H (RNase H) activity, a ribonuclease that dismantles RNA strands in RNA–DNA hybrids. When R-loops form during transcription, they create a structural barrier that can stall the progression of RNA Pol III along the DNA strand. This stalling can interrupt the production of tRNAs and other small RNAs, potentially impairing protein synthesis [32]. Additionally, forming these R-loops during RNA Pol III transcription can lead to conflicts between the transcription and replication machinery. These unscheduled R-loops can result in genome instability, such as replication stress and DNA breaks, triggering various cellular responses, including DNA repair, cell-cycle arrest, and apoptosis [23,24,34,35]. Unlike RNA Pol I, RNA Pol III genes are scattered throughout the linear chromosome maps, in which the spatial organization of these genes affect a substantial portion of other genes in the genome [36]. Thus, the transcriptional stalling of RNA Pol III may have more widespread, adverse genomic effects.

### 2.2. R-Loops during Replication

A notable area of interest revolves around the biological function of R-loops in DNA replication. When DNA replication coincides with ongoing transcription in the S phase, TRCs arise and impede DNA synthesis [35,37]. R-loops, the most prominent source of R-loop-mediated damage during the S phase, seem to deteriorate TRCs and potentially cause lethal DNA damage and threaten cell survival [38,39,40]. R-loops have been shown to interfere with DNA replication by inhibiting the progression of replication forks under hormone or oncogene-induced replication stress [41,42,43]. Using electron microscopy and immuno-labeling techniques, one recent study provides direct evidence of R-loop formation and its association with RNA–DNA hybrids behind replication forks, causing fork slowing and reversal and shedding light on the mechanisms underlying TRC-associated replication interference [43]. To precisely coordinate the harmful conflicts, overexpressing RNA–DNA endonuclease RNase H1 can remove DNA damage produced by R-loops and recover DNA synthesis [44,45]. The ATP-dependent chromatin remodeling complex INO80 plays a pivotal role in resolving R-loops linked to DNA damage during replication in cancer cells. Furthermore, R-loops interact with and facilitate INO80 recruitment to chromatin, and the artificial tethering enhances R-loop turnover. Ultimately, INO80-mediated R-loop resolution supports DNA replication and transcription, fostering proliferation and safeguarding against DNA-damage-induced cell death in cancer cells [46]. Understanding the dynamics of R-loop formation and resolution during the S phase, and finding ways to prevent their excessive accumulation, is crucial to maintaining genomic stability during DNA replication [37].

### 2.3. R-Loops in Genome Editing

R-loops not only inadvertently form during transcription as the main genomic instability source, but also intentionally form as a tool for regulating transcription and DNA metabolism [5]. R-loops have gained substantial attention in the context of genome editing due to their intrinsic ability to highlight targeted genomic regions [47,48]. Remarkable genome editing tools, such as CRISPR-Cas9, which rely on accurate and specific targeting, can benefit significantly from the guiding properties of R-loops. The single guide RNA (sgRNA) initiates these R-loops by meticulously aligning with a matching sequence in the genome, steering the Cas9 nuclease toward its predetermined genomic destination [48,49]. Upon arrival, the Cas9 nuclease induces a DSB in the specific gene site, triggering cellular repair mechanisms, such as non-homologous end joining (NHEJ) or HR, leading to the desired genetic modifications [48,50,51]. The presence and stability of R-loops at both intended (on-target) and unintended (off-target) genomic sites have a profound impact on the precision and efficiency of genome editing [52]. The Cas9–sgRNA complex divides the target DNA into distinct domains and is identified as an intermediate state preceding the stable R-loop formation, shedding light on the R-loop formation process and the factors contributing to off-target effects in the CRISPR/Cas9 system [53]. In addition, Cas9 undergoes conformational changes in the early phase of R-loop formation as the guide RNA and target DNA hybridize, leading to HNH nuclease inactivation in Streptococcus pyogenes. However, when the heteroduplex forms completely, it activates the HNH nuclease, providing insights for designing more effective Cas9 variants and guide RNAs to minimize off-target effects [54].

A recent study reveals that site-specific R-loop formation is necessary and sufficient in a potential therapeutic approach for fragile X syndrome (FXS), a common cause of autism spectrum disorders. FXS results from the epigenetic silencing of the FMR1 gene due to the expansion of a trinucleotide repeat (CGG). MEK and BRAF inhibitors induce DNA demethylation, R-loop formation, and repeat contraction at the FMR1 gene sites, which subsequently recruit endogenous DNA repair factors to excise the expanded CGG repeats. These inhibitors have great potential to serve as a promising tool for FXS treatment [55].

### 2.4. R-Loops at DNA Damage Sites of Transcriptionally Active Loci

R-loops have been detected at DNA damage sites, particularly in transcriptionally active regions, and maintaining the stability of R-loops plays a crucial role by preserving genomic stability [56,57]. When the transcriptional machinery comes across a location of DNA damage, the transcriptional apparatus may stall, triggering the development of an R-loop structure. The R-loop can subsequently serve as a marker for the DNA damage response system, aiding in the recruitment of repair factors to the damage sites [6,58]. Within transcriptionally active areas, these configurations can both enhance and hinder DNA repair [6]. On one hand, R-loop formation serves as a protective mechanism for DNA, mitigating any additional DNA damage and facilitating the mobilization of DNA repair proteins to the site of injury. RNA–DNA hybrids have been observed at sites of DSBs and single-stranded breaks (SSBs), which are highly dependent on local transcription [57,59].

Conversely, establishing R-loops at DNA damage sites can escalate genomic instability via multiple processes [60]. The RNA–DNA hybrid can physically obstruct the repair machinery, disrupting the repair process [61,62]. Moreover, unresolved R-loops can instigate conflicts with the replication apparatus, inducing replication stress and potentially producing DNA ruptures. Although R-loops have vital roles in DNA damage response within transcriptionally active loci [63], their exact functions and regulations necessitate further studies [56]. Investigating R-loops’ regulatory mechanisms and their impacts on DNA repair and genome stability is a growing field of research.

### 2.5. Mitochondrial R-Loops

R-loops are also predominantly located in the major regulatory regions of the mammalian mtDNA [64]. Mitochondria, the powerhouses of eukaryotic cells, possess independent small, circular DNA, coined mtDNA [64]. The transcription of mtDNA is indispensable for synthesizing proteins vital for the energy production processes in cells [17]. During transcription, R-loops tend to form when the newly synthesized RNA molecule binds with the DNA template strand, resulting in an unpaired non-template strand [65]. Just as R-loops influence gene expression, replication, and genomic stability in the nuclear genome, mitochondrial R-loops exhibit similar impacts. Previous studies reveal that forming R-loops may regulate mtDNA replication and transcription [16,66]. R-loops may also inhibit mtDNA strand separation at proximal sites and prevent transcription at promoter sites [16,17]. However, the mechanisms by which R-loops affect mtDNA transcription is still not fully understood. Furthermore, mitochondrial R-loops could affect the regulation of mtDNA copy number. R-loops regulate mtDNA replication and thus mtDNA copy number by exposing the primer start site for the initiation of leading-strand replication. Moreover, RNA strands in RNA–DNA hybrids serve as a primer for the initiation of mtDNA replication at original replication sites [16,17,67,68]. However, the overaccumulation of R-loops can lead to clashes between the transcription and replication machinery, which can cause replication stress, DNA damage, and possible genomic instability [69]. Hence, cells have evolved mechanisms to prevent R-loops’ overaccumulation. For instance, the endonuclease RNase H1, found in both the nucleus and mitochondria, can dismantle R-loops by degrading the RNA strand of RNA–DNA hybrids [66,70]. Using next-generation sequencing, mutations in the RNase H1 gene were identified in individuals and affected by chronic progressive external ophthalmoplegia (CPEO), a mitochondrial disorder. These mutations, either compound heterozygous or homozygous, result in dysfunctional RNase H1 functions, shedding light on the pathogenic mechanisms underlying CPEO and emphasizing the significance of RNase H1 in maintaining mitochondrial DNA integrity [66]. In addition, the degradome complex in the mitochondria, composed of the SUV3 helicase and the ribonuclease polyribonucleotide nucleotidyltransferase 1, plays a critical role in averting the buildup of detrimental R-loops in the mitochondria [66,69,71].

### 2.6. R-Loops in Telomeres and Sub-Telomeres

Telomeric repeat-containing RNA (TERRA) represents a sophisticated class of long non-coding RNAs (lncRNAs), characterized by its UUAGGG repeats. Intriguingly, these sequences engage in a hybridization process with the C-rich strand of telomeric DNA, undergoing transcription under the action of RNA polymerase II within both telomeric and adjacent sub-telomeric domains [72,73,74]. In human cells, TERRA interacts with several telomere-binding proteins and chromatin modulators, such as telomere repeat-binding factor 2 (TRF2) and ATRX, in which TERRA is vital for carrying out telomere maintenance [74,75]. It has been revealed that TERRA-generated R-loops are involved in promoting telomerase recruitment at short telomeres [76]. Recently, a study revealed that RNA containing the UUAGGG repeats can form telomeric R-loops in trans via a RAD51 recombinase-mediated mechanism [77]. Moreover, the researchers found that telomeric R-loops lead to heightened telomere fragility, which can be counteracted by RNaseH1 and TRF1 recruitment [76,77]. Therefore, telomeric R-loops allow TERRA to associate with and to maintain telomeres, but they also result in genomic instability, which is likely crucial for the proper functions of telomeres [72,76,77,78].

The endonuclease XPF, a TERRA-interacting protein, is highly enriched at alternative lengthening of telomeres (ALT) and is recruited by telomeric R-loops to induce DNA damage response (DDR) independent of CSB and SLX4, giving rise to break-induced telomere synthesis and lengthening [79]. The recruitment of BRCA1 and RAD51 to telomeres requires XPF in FANCM-deficient cells with accumulated telomeric R-loops, suggesting that telomeric R-loops activate DDR via XPF to promote homologous recombination (HR) and telomere replication to trigger ALT [79,80]. It is crucial to regulate TERRA and telomeric R-loops properly for the optimal function of telomeres.

### 2.7. R-Loops in Centromeres

Centromeres epitomize a specialized chromatin landscape, which are orchestrated by incorporating the histone H3 analog, centromeric protein A (CENP-A), onto the recurring α-satellite motifs. Notably, this genomic architecture undergoes consistent transcriptional activity mediated by RNA Pol II throughout the cell cycle [81,82]. In human cells, centromeric and pericentric satellite RNA transcripts are readily detected [83]. The RNA transcripts of α-satellite repeats are associated with centromeres [84,85], suggesting that there are R-loops in cis in mitotic chromosomes [83]. It is shown that BRCA1 associates with centromeric chromatin depending on the presence of R-loops, in which BRCA1 counteracts the accumulation of R-loops at centromeric α-satellite repeats [82]. Centromeric RNA is the crucial bridge for CENP-A and CENP-C with centromeres, rendering these RNA transcripts as vital structural components of centromeres [84,85]. During the S phase, CENP-A is necessary to prevent transcription and R-loops at centromeres, protecting centromeres from DNA damage [84,86].

Previous research illuminates that the serine/threonine protein kinase ATR exhibits targeted recruitment to centromeres amid mitosis, contingent upon R-loops’ presence. This strategic positioning empowers ATR to facilitate the activation of Aurora B, mediated through an intricate liaison with CHK1 [5,83]. However, although centromeric R-loops are essential for centromere assembly and functions, they are also a curial source of replication stress. Hence, centromeric R-loops and associated proteins play a double-edged-sword role and must be intricately orchestrated during the cell cycle [2].

### 2.8. Cytoplasmic R-Loops and RNA–DNA Hybrids

Cytoplasmic R-loops and RNA–DNA hybrids participate in multiple biological processes, including RNA metabolism, immune responses, and pathological conditions [87]. Cytoplasmic RNA–DNA hybrids can be found in naturally aged liver, kidney, and pancreas cells, stemmed from nuclear R-loop processing, which possess immunogenic properties [88]. Notably, recent evidence unveils a population of RNA–DNA hybrids in the cytoplasm resulting from disturbances in nuclear R-loops, induced by depleting key proteins like Senataxin (SETX) and BRCA1 [89,90]. These cytoplasmic hybrids, originating from specific stable nuclear R-loops, activate pattern recognition receptors like cGAS and TLR3, triggering an immune response and cell apoptosis through IRF3 activation. Moreover, cytosolic RNA–DNA hybrids activate the cGAS–STING [87,91] pathway in THP-1 knockout cells, which is a critical pathway promoting the induction of an innate immune response. Accumulation of these immunogenic cytosolic hybrids are linked to pathological conditions, suggesting that aberrant R-loop processing and subsequent immune activation may be pathological processes that differentially affect human diseases. Cytosolic hybrids in patients with SETX-mutated ataxia oculomotor apraxia type 2 (AOA2) and BRCA1-mutated cancer cells initiate a IRF3-mediated immune response that triggers apoptosis [89]. These findings suggest that RNA–DNA hybrids are immunogenic, and their abnormal accumulation in the cytoplasm due to R-loop processing links R-loop dysregulation to cell death via innate immune activation, potentially contributing to diseases like neurodegeneration and cancer [92]. The regulation of these cytosolic hybrids is still not fully understood; however, it has been observed in various human cancer cell lines that Exportin-1, a key protein that mediates nuclear export, mediates transport of RNA–DNA hybrids to the cytosol and RNA Pol III regulates the presence of cytosolic RNA–DNA hybrids [88]. Inhibition of Pol III was found to be effective in preventing the formation of cytosolic RNA–DNA hybrids. These hybrids interact with components of the microRNA machinery and are linked to specific miRNA regulation, revealing a role for Pol III in regulating these hybrids and miRNA biogenesis in human cells [93]. Nonetheless, these findings suggest that abnormal accumulation of RNA–DNA hybrids in the cytoplasm elicit innate immune response and cell death, potentially contributing to diseases like neurodegeneration and cancer.

## 3. Suppression and Resolution Mechanisms of R-Loops

R-loops can form in a regulated process mediated by specific protein factors or spontaneously in an unscheduled manner. Cells employ a symphony of finely tuned mechanisms to judiciously oversee the genesis and resolution of R-loops, ensuring precise gene regulation while bypassing inadvertent mutagenic calamities [1,2,94]. DDX1 helicase unwinds G-quadraplex (G4) structures in IgH transcripts, allowing RNA to hybridize with the DNA template, forming an R-loop structure and regulating class-switch recombination [95]. Moreover, DHX9 RNA helicases promote physiologic R-loops’ formation at centromeres by unwinding RNA secondary structures [96]. Though these specific protein machineries have yet to be fully understood, they serve as evidence that physiological R-loops serve an important role in controlling gene expression, which stands in stark contrast to unscheduled, spontaneous R-loops’ formation in which cells readily resolve via multiple mechanisms to tightly control the formation of R-loops. Several proteins modulate the resolution of unscheduled R-loops either directly or indirectly to limit their harmful consequences towards genome stability. The overarching objective is to prevent the RNA from engaging in hybridization with DNA, thereby adverting the spontaneous accumulation of unscheduled R-loops [6,12,44,97]. Important factors involved in R-loops suppression and resolution are summarized below and in Table 1.

### 3.1. RNase H1 and RNase H2

In the complicated framework of nucleic acid metabolism, RNase H precisely stands out, representing a ubiquitous ribonuclease whose lineage traces from rudimentary bacteria to sophisticated humans. This enzyme precisely dismantles the RNA components of RNA–DNA hybrids, epitomizing its specificity in function [44,97,98]. Acting as endoribonucleases, RNase H proteins are vital for nucleic acid homeostasis, meticulously catalyzing RNA within RNA–DNA hybrids [44]. Their important roles in preventing the accumulation of unscheduled R-loops—potential contributors to genomic instability—cannot be overstated. Notably, mutations in the normal function or structural integrity of RNase H proteins are intricately linked with a multitude of human pathologies, underscoring their importance in cellular processes. Diving deeper, both RNase H1 and RNase H2 emerge as important enzymatic regulators that catalyze RNA presence in RNA–DNA hybrids, maintaining genomic stability and preventing R-loop proliferation [99]. Meanwhile, RNase H2 predominantly functions during the G2/M cell-cycle checkpoint, orchestrating both R-loops’ processing and ribonucleotide repair, RNase H1 operates independently across the cell cycle, being particularly recruited during elevated R-loop concentrations [97].

RNase H1, an endonuclease involved in the degradation of RNA–DNA hybrids, is predominantly localized within the mitochondria, and to a more nuanced extent, within the cell nucleus. This enzyme acts as a defense mechanism, eradicating R-loops and ensuring a harmonious interplay between transcriptional and replicative processes. Mutations in its regulatory structure tether it to a spectrum of mitochondrial pathologies and oncological conditions [44,97]. A depletion of RNaseH1 orchestrates a cascade of cellular events: the accumulation of telomeric hybrids, the unwinding of single-stranded telomeric DNA, the activation of replication protein A(RPA), and the unscheduled excision of telomeres. Contrarily, an amplified expression of RNaseH1 reduces the recombinogenic potential of ALT telomeres, culminating in telomeric shortening. These findings highlight the roles of RNaseH1 in preserving an equilibrium of telomeric RNA–DNA hybrids, essential for HR-mediated telomere sustenance in ALT cells, all while preserving telomeric integrity [76]. Leveraging expansive datasets from The Cancer Genome Atlas (TCGA) and the Genotype-Tissue Expression (GTEx) compendium, RNase H1’s expression across diverse cancers has been mapped. In a staggering 19 malignancies linked to unfavorable prognosis, RNase H1’s overexpression stood out. Furthermore, its expression portrayed a correlative connection with tumor microenvironment modulation, immune cellular infiltration, and activities synchronous with DNA and mitochondrial dynamics. These findings position RNASEH1 as a beacon for potential oncological biomarker exploration and paves the way for its consideration in developing innovative therapeutic avenues [100].

RNase H2, primarily located within the nucleus, showcases broad substrate specificity, adeptly resolving not just RNA–DNA hybrids, but also singular ribonucleotides embedded within DNA. Its journey from the cytosol to its nuclear locations is instrumental, facilitating its recruitment at critical junctures of DNA replication and restoration. Defective RNase H2 draws a tangible link to Aicardi–Goutieres Syndrome (AGS), systemic lupus erythematosus, and an amplified oncological susceptibility, promoted by the disruption of complex assemblies and the disorder of ribonucleotide removal [101,102,103,104]. Furthermore, a previous study reconstituted the ribonucleotide excision repair (RER) complex, utilizing purified enzymes extracted from Saccharomyces cerevisiae. This reconstitution highlights the mechanism of RNase H2 in cleaving the ribonucleotide, setting the stage for FEN1-mediated ejection, followed by synthesis conducted by DNA polymerase δ, PCNA, RFC, and DNA ligase I. While a handful of enzymes within the complex exhibit redundancy, RNase H1 falls short of reproducing RNase H2 functions in the incisive phase of RER [99].

Beyond their similar function of maintaining genomic stability, these crucial enzymes, with their distinctive substrate affinities and cellular domains, exhibit independent roles in cellular mechanics. Deciphering the precise role of these enzymes can potentially highlight the complex etiology of pathological conditions for therapeutic avenues.

### 3.2. RNA–DNA Helicases

RNA–DNA helicases are enzymes that play vital roles in the S phase of DNA replication, transcription, and repair by unwinding and separating the DNA double helix. They are crucial in separating RNA molecules from DNA duplexes and displacing proteins and nucleic acids bound to DNA or RNA strands. Using the energy from ATP hydrolysis, they can unwind the hydrogen-bonded base pairs, creating DNA regions that are accessible to cellular machinery like DNA polymerases or RNA polymerases [105,106]. RNA–DNA helicases, such as Aquarius (AQR) and SETX, are responsible for unwinding and dissociating the RNA–DNA hybrid structure, actively promoting the resolution of R-loops to maintain genomic integrity and facilitating various cellular processes [107,108,109].

The DEAD (Asp-Glu-Ala-Asp) box cohort of RNA helicases, similar to the RNA-binding protein (RBP) complexes, carries out various metabolic processes in RNA metabolism in transcription, mRNA translocation, and RNA catabolism [110,111,112]. The pleiotropic capabilities of certain DEAD-box family constituents orchestrate regulation of transcriptional activity and the equilibrium of R-loop formation [113]. Notably, the DDX21 helicase, located within the nucleolus, is a crucial enzyme for the conversion of 20S rRNA to its 18S partner. Intriguingly, a depletion in DDX21 expression catalyzes a notable surge in R-loop genesis with hallmarks of RNA Pol II hindrance and proliferation of γH2AX foci, thereby underlining the critical roles of DDX21 in the catalysis of R-loops and as a defense mechanism against genomic instability [113]. Furthermore, DDX21’s versatility extends to reduced estrogen-facilitated R-loops genesis in mammary oncogenic cells with reduced replication-induced stress in neural crest progenitors and melanoma cellular entities [113,114].

Similarly the DEAD-box helicase DHX9 is integral to transcriptional activity by facilitating the interactions between RNA Pol II, the transcriptional co-activator p300 [115], and tumor suppressor BRCA1 [116]. DHX9 also showcases adeptness in resolving RNA–DNA hybrids, as well as more intricate nucleic acid configurations such as G-quadruplexes [117]. In addition, a potentially joint relationship between DHX9 and PARP1 has been shown to preempt R-loop-mediated DNA damage [118].

The Bloom helicase (BLM) occupies an instrumental niche within the R-loop processing landscape, accurately resolving RNA–DNA hybrids, thereby degrading the interaction between the RNA moiety and its DNA counterpart [119,120]. Such molecular maneuvers serve as defense mechanisms against the harmful consequences of unscheduled R-loops, including DNA lesions, replicative stress, and genomic instability. Previous studies using budding yeast models unveil that the deficiency of the BLM ortholog, Sgs1, predisposes cells to a perilous interface between replication and transcription, promoting unscheduled R-loop formation. Unscheduled R-loop genesis contributed to the escalation in DNA damage and dysregulation in genomic copy number equilibria [121]. Additionally, multiple studies illustrate that at the telomeric ends of ALT cells, BLM and BRCA1 play an important mechanistic role. Here, BRCA1 stimulates BLM unwinding activity on the telomeric fork, allowing BRCA1 to carry out recombinational processes, ultimately promoting telomere extension and ALT cellular longevity [119,122]. These studies highlight the importance of the BLM–BRCA1 complex in telomere extension, a potential therapeutic opportunity for malignancies in which ALT is implicated [123].

Moreover, it is recently reported that FANCM plays a crucial role in restricting alternative telomeres’ lengthening by mitigating telomeric replication stress induced by dysregulated BLM helicases and R-loops [124]. Dysregulation of BLM helicase has been shown to trigger the formation of R-loops at telomeres. However, FANCM acts as a protective factor by unwinding these R-loops, preventing their accumulation, and reducing ALT activity [80,120]. Such insights deepen our understanding of the mechanisms of FANCM on telomeric R-loops and ALT kinetics, offering glimpses into targeted therapeutic strategies for ALT-associated cancers.

### 3.3. Chromatin Remodeling Factors

Chromatin remodeling factors represent a heterogeneous ensemble of proteins pivotal in regulating chromatin structure and modification. Engaged in various cellular processes, including regulation of transcriptional activity, DNA replication, and DNA repair, chromatin remodeling factors function via nucleosome remodeling and histone modification. These DNA alterations facilitate shifts in gene accessibility, ultimately influencing transcriptional activity [22,125,126].

There is an intricate relationship between chromatin remodeling factors and the formation and resolution of R-loops. Chromatin remodeling factors can influence R-loops formation by modulating the accessibility of DNA regions and the recruitment of factors involved in RNA processing [22]. One study reveals that the depletion of facilitates chromatin transcription (FACT) complex leads to replication impairment and the accumulation of R-loops, which can be alleviated by overexpression of RNase H1 or inhibition of global RNA synthesis [127].

It is also revealed that ATP-dependent chromatin remodelers, such as the SWI/SNF complex, are frequently mutated in cancer and play a crucial role in maintaining genomic stability. Depletion of the BRG1 subunit of SWI/SNF leads to increased R-loops’ formation, R-loop-dependent DNA breaks, and transcription–replication conflicts, highlighting the importance of the SWI/SNF complex, particularly the cBAF variants, in resolving R-loop-mediated conflicts and safeguarding genomic integrity [128].

Moreover, chromatin remodelers, such as alpha thalassemia/mental retardation syndrome X-linked (ATRX) [129] and chromodomain helicase DNA-binding protein 4 (CHD4), have been implicated in suppressing R-loops’ accumulation and promoting their resolution [130]. Furthermore, chromatin remodeling factors contribute to the regulation of R-loop-mediated processes. It is reported that BRCA1, a well-known chromatin remodeling protein involved in DNA repair, has been found to play a role in suppressing R-loops [131]. BRCA1 interacts with RNA–DNA hybrids and promotes R-loop resolution, preventing potential genomic instability and DNA damage [132]. Many other chromatin remodeling factors, such as AID/APOBEC enzymes, have also been implicated in modulating R-loop levels and influencing gene expression patterns [133].

Gaining insights into the relationship between chromatin remodeling factors and R-loops is crucial for understanding the mechanisms that govern genomic stability and gene expression. Disturbances in these processes can result in genomic instability, DNA damage, and the onset of diverse diseases, including cancer. Further research is needed to elucidate the precise mechanisms through which chromatin remodeling factors influence the dynamics of R-loops and their implications in normal cellular functions and the development of pathological disease.

### 3.4. DNA Repair Proteins

Various DNA repair proteins have been found to play crucial roles in suppressing R-loops by actively resolving and preventing their accumulation. Within the gamut of DNA repair mechanisms, the nucleotide excision repair (NER) pathway stands out, accurately locating and resolving DNA mutations, R-loops included, via an astoundingly sophisticated process. The excision repair machinery is precisely recruited to R-loop formations to excise the RNA strand embedded within the DNA matrix [109]. This process promotes the recruitment of specialized proteins like XPG and XPF to the R-loop locus [31], which then coordinates the excision and repair of the damaged DNA. Moreover, the Fanconi anemia (FA) repair pathway has been established to protect cells from R-loops, contributing to genomic instability and DNA damage [134,135,136]. It is verified that the FANCI–FANCD2 complex plays a vital role in preventing the formation of harmful R-loops by binding to single-stranded RNA and DNA molecules, leading to the activation of the FA pathway [137].

One study reveals that RPA, an ssDNA-binding protein, interacts with and regulates the activity of RNaseH1, an enzyme involved in R-loop suppression [138]. Moreover, one study reveals that BRCA1 associates with TTSs and recruits SETX to suppress R-loop formation. In breast luminal epithelial cells with BRCA1 mutation carriers, BRCA1 deficiency leads to R-loop accumulation because of RNA Pol II pausing-mediated R-loop formation [82,90,116,131]. Similarly, BRCA2 deficiency is associated with elevated levels of RNA Pol II pausing and the accumulation of R-loops at sites proximal to gene promoters. In a complex mechanism, BRCA2 interacts with the TREX2 complex to inhibit R-loop formation and recruits DDX5 to suppress R-loops in transcribed regions. These observations reveal that BRCA2 regulates R-loop dynamics and provides insights into how BRCA2 prevents R-loop-mediated genomic instability. Their functions include recognizing and removing the RNA component of the R-loop, promoting DNA repair and preventing the accumulation of DNA damage that can arise from unresolved R-loops [139,140,141,142].

It is crucial to manage R-loops and prevent unscheduled accumulation properly. Various suppression mechanisms work synergistically to achieve this. Any failure in these mechanisms can cause an increase in R-loop levels, which could lead to DNA damage and genomic instability. Such instability may also contribute to developing diseases like cancer and neurodegenerative disorders. Therefore, it is vital to keep these mechanisms in check to prevent any unwanted consequences.

**Table 1:** Factors related with R-loop suppression and resolution.

Type of Protein	Functions	References
Topoisomerase		
TOP1	A class of loci overlapping with efficient early replication origins showed an unexpected loss of R-loops upon Top1 depletion	[143,144]
TOP3B	TOP3B works in an epistatic manner with the helicase DDX5 to resolve cellular R-loops	[145]
Ribonucleases		
RNaseH1	Cleaves RNA in RNA–DNA hybrids	[7,98]
RNaseH2	Cleaves RNA in RNA–DNA hybrids; Removes ribonucleotides	[99,146,147]
RNA–DNA helicases		
DDX1	Associates with ATAD5 and suppresses R-loops during DNA replication	[148]
DDX2A/ DDX2B	Unwinds the 5′ leader structure as part of the eIF4F complex; reduces affinity of eIF3j for the ribosome to enable the mRNA to access the entry channel	[149]
DDX3X	Pre-mRNA splicing; general mRNA export	[150,151]
DDX5	Associates with BRCA2 and suppresses R-loops at DSBs; together with ATAD5, it suppresses R-loops during replication	[140,148,152]
DDX19	Enters the nucleus following DNA damage in an ATR-dependent manner; suppresses R-loops	[153]
DDX21	Unwinds RNA–DNA hybrids in vitro; functions with SIRT7 to suppress R-loops at specific genes; associates with ATAD5; suppresses R-loops during replication	[113]
DDX23	Involved in mRNA processing by regulating of FOXM1	[154,155]
DDX39B(UAP56)	Unwinds RNA–DNA hybrids in vitro; suppresses co-transcriptional R-loops	[156,157,158,159]
DDX41	Enriched in promoter regions in vivo; unwind RNA–DNA hybrids in vitro	[160,161]
DDX43	ATP-dependent; aids piRNA amplification by liberating cleaved RNAs from Ago3-piRISC	[162,163]
DHX9	Binds RNA–DNA hybrids and unwinds R-loops; associates with ATAD5 and suppresses R-loops during replication; prevents R-loop formation by melting RNA–DNA hybrids with a 3′–5′ polarity	[96,117,118,164]
DHX29	Rearrangement of 43S complexes leading to higher processivity in unwinding during scanning	[165,166]
DHX36	Regulates the translation of Gnai2 mRNA by unwinding its 5′ UTR rG4 structures	[167]
WRN	Protects the replication fork by preventing unprogrammed R-loops formation	[168]
AQR	Putative RNA–DNA helicase; suppresses R-loops in human cells	[109]
BLM	Unwinds RNA–DNA hybrids in vitro; suppresses R-loops in human cells	[169,170]
SETX	Suppresses R-loops at transcription termination sites and DSBs; binds to replication forks to protect its integrity across RNA-Polymerase-II-transcribed gene and unwinds unnecessary R-loops	[63,171]
RECQL4	Resolves concatenated DNA molecules during HR, at replication forks, and in anaphase	[172,173,174]
FANCM	Unwinds telomeric RNA–DNA hybrids; suppresses telomeric R-loops in human cells	[80,120,124]
DNA-damage checkpoint proteins		
ATR, CHK1	May suppress R-loops by preventing collisions between R-loops and replication forks	[153,175]
ATM, CHK2	May suppress R-loops by promoting the repair of DSBs at replication forks collapsed at R-loops	[176,177]
Chromatin modulators		
SWI/SNF	ATP-dependent chromatin-remodeling complexes, may suppress R-loops during transcription–replication conflicts	[128,178]
PCR1	Promotes repressive chromatin, suppresses R-loops by decreasing transcription	[179]
BRD2	A reader of histone lysine acetylation; recruits TOP1	[180]
BRD4	A reader of histone lysine acetylation; suppresses R-loops at specific genes by preventing Pol II pausing	[39,130]
FACT	Histone chaperone, may reorganize chromatin at sites of R-loop-replication collisions	[127]
KAT8	A histone acetyltransferase that functions with c and BRD4 to suppress R-loops	[180]
SIN3A	Part of a histone deacetylase complex that interacts with the THO complex	[181]
INO80	Part of the ATP-dependent chromatin-remodeling complex, may reorganize chromatin to resolve R-loops	[46]
DNA repair proteins		
BRCA1	Suppresses R-loops at promoter-proximal Pol II pause sites and transcription termination sites; recruits SETX to transcription termination sites	[82,90,182,183]
BRCA2	Suppresses R-loops at promoter-proximal Pol II pause sites; interacts with DDX5 and TREX2	[139,140,141,142]
FA factors(A/I/D2/L) Likely entire pathway	FANCD2–FANCI bind RNA–DNA hybrids and RNA processing factors suppress R-loops to coordinate replication and transcription; the repair of RFs blocked at R-loop-containing sites	[135,136,137]
RPA	Recruits, binds, and stimulates RNaseH1	[138]
CtIP	May process R-loops at active genes	[184]
APTX/TDP1	Suppresses R-loops through its function in single-strand break repair	[185]
MRE11	Suppresses R-loops upon collisions of R-loops and replication forks, promotes functions of FA repair proteins	[186]
MUS81	A structure-specific nuclease that cleaves Holliday junctions and stalled or reversed replication forks, may process R-loops upon collisions of R-loops and replication forks	[44,175]
SAMHD1	Deoxynucleoside triphosphohydrolase (dNTPase) and 3′–5′ exoribonuclease; does not resolve R-loops directly but can recruit other factors like MRE11	[187]
XPG, XPF	Structure-specific nucleases involved in transcription-coupled nucleotide excision repair, may cleave and remove R-loops during transcription	[31,79]
RNA processing factors		
SRSF1	Splicing factor; binds the Pol II CTD and nascent RNA transcripts	[188]
SRSF2	Splicing factors; mutated in myelodysplastic syndrome and cancer	[189]
SF3B1	Splicing factors; mutated in myelodysplastic syndrome and cancer	[190,191]
SPT6	Pol II-associated elongation factor recruits the Integrator complex to suppress R-loops generated from long non-coding RNA	[192]
TFIIS	Recognizes backtracked Pol II and stimulates transcript cleavage	[193]
U2AF1	Splicing factors; mutated in myelodysplastic syndrome and cancer	[191]
SFPQ, NONO	RNA splicing; suppress telomeric R-loops	[194]
RNA exosome	3′–5′ exoribonuclease; degrades non-coding RNAs and prevents R-loop accumulation	[195,196]
THO/TREX/TREX2	Promote transcription termination and assembly and nuclear export of messenger ribonucleoproteins	[40,141,181,197]

## 4. R-Loops as a Double-Edged Sword

There has been increasing evidence that R-loops act as a double-edged sword in biological life over the past few years. R-loops play an instrumental role in transcriptional regulation and DNA repair, yet unregulated R-loop production has been found to promote genome instability. Thus, cells must exploit the functions of R-loops in a controlled and regulated manner. Here, we analyze R-loops as important cellular regulators and their potential to generate genomic threats.

### 4.1. Functions of R-Loops

#### 4.1.1. R-Loops and Transcriptional Regulation

**Figure 1:**
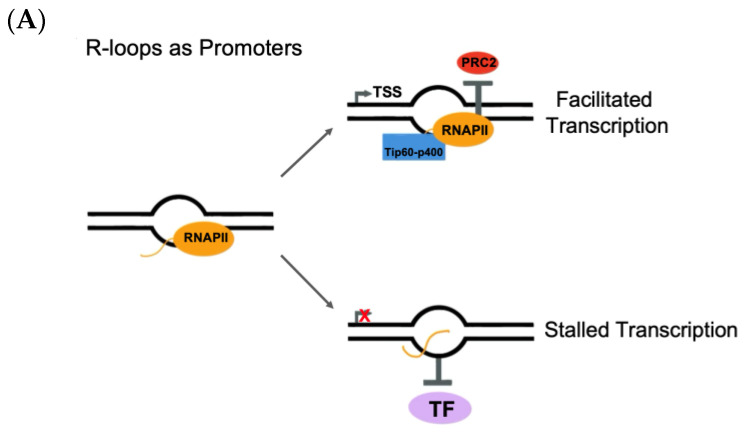

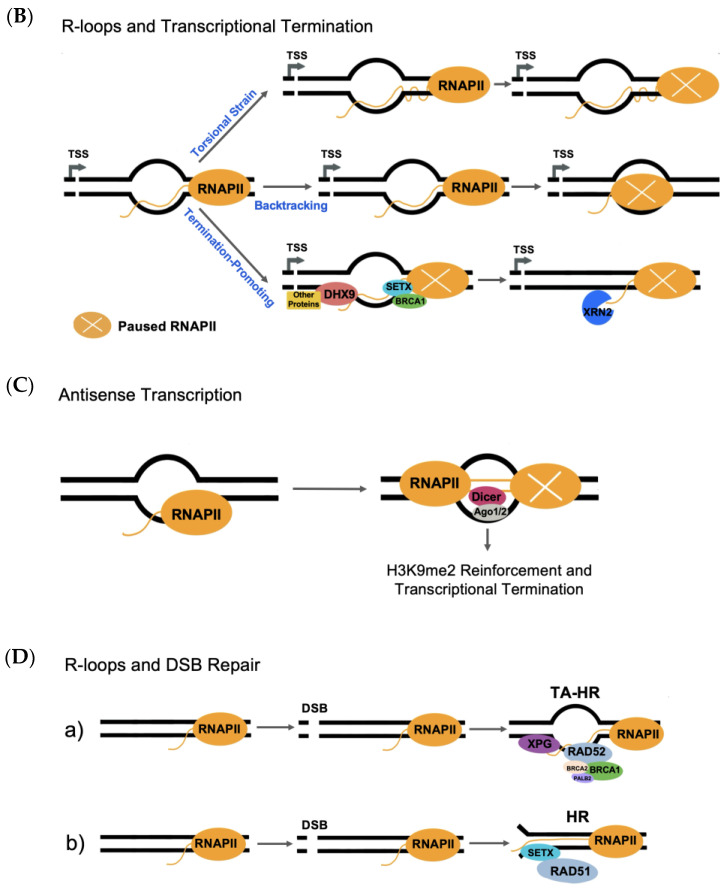
Functions of R-loops. (**A**) R-loops act as a promoter. R-loops formation promotes the binding of tip60-p400 which activates chromatin for transcription. Moreover R-loop formation inhibits the binding of PRC2 which silences chromatin via DNA methyltransferase. TSS: transcription start site. TF: transcription factors. Red cross mark: stalled transcription. (**B**) Three mechanisms of R-loops as transcriptional termination; (**C**) R-loops promote antisense transcription to facilitate transcriptional termination; (**D**) R-loops in DSB repair: (a) DSBs promote R-loops formation. As the R-loop is formed, it recruits RAD52 and BRCA1. BRCA1 recruits BRCA2 and PALB2 which promotes the removal of the RNA from the RNA-DNA hybrids. XPG is then recruited to incise the R-loop which in turn promotes TA-HR. (b) DSBs promote the recruitment of SETX and RAD51 to promote HR at the RNA-DNA hybrid.

R-loops function as promoters regulating transcription and thus gene expression. Despite being detected throughout the whole genome, R-loops have been found in highly transcribed regions, particularly at RNA Pol II transcription start sites (TSSs) [198]. R-loops regulate gene expression through multiple context-dependent mechanisms. R-loops have been shown to regulate two chromatin binding complexes, Tip60-p400 and polycomb repressive complex 2 (PRC2) [125]. Tip60-p400 has been observed to bind to chromatin when transcription is activated, unlike PRC2, in which transcription deactivates the chromatin binding of PRC2 [125,199]. At the TSS, R-loops facilitate transcription by protecting DNA from the binding of PRC2 [125]. PRC2 methylates the DNA it binds to, subsequently promoting methylation-associated silencing via DNA methyltransferase [200]. Moreover, R-loops promote chromatin binding of Tip60-p400, thus activating transcription. In vivo models of mouse embryonic stem cells (ESCs) exhibit R-loops inhibition, deactivating chromatin enzymes, such as PRC2, and promoting the binding of activating chromatin remodeling complexes like Tip60-p400 to facilitate a poised chromatin state. (Figure 1A) [125]. Additionally, R-loops can block binding of transcription factors, though this mechanism has only been seen at specific promoter loci (Figure 1A) [201]. In budding yeast cells, R-loops that stem from long non-coding regions of RNA alter chromatin architecture by displacing co-repressors and promoting transcription of nutrient utilization genes [202].

#### 4.1.2. R-Loops and Transcriptional Termination

R-loops also play a critical role in transcription termination of certain mammalian genes. Transcription termination is an extremely intricate process for protein coding genes, and any failure in this process affects gene expression [203]. Thus, R-loop formation and dismantling must be carefully regulated to ensure gene expression. R-loops have been found to reside in the 3′ end of protein coding genes to regulate transcription termination [204].

Primarily, R-loops are known to pause RNA Pol II to facilitate transcription termination. RNA Pol II pausing can be produced by hybridization of the nascent transcript with the antisense DNA strand, resulting in the formation of R-loops [7]. Multiple mechanisms are evident by which R-loops stall RNA Pol II. One such mechanism terminates transcription via torsional strain. During elongation, RNA Pol II rotates around the helical path and thus produces the nascent transcript to wrap around the DNA upstream of the R-loop. This wrapping of the nascent transcript around the DNA is energetically unfavorable and initiates transcription termination (Figure 1B) [205]. Another such mechanism is backtracking termination. RNA Pol II transcribes in both forward and backward directions because when RNA Pol II backtracks, it functions as a proofreading mechanism [206]. RNA loops have been found to function as an exit channel for RNA Pol II when RNA Pol II backtracks, effectively terminating RNA Pol II and thus transcription (Figure 1B) [7]. Lastly, RNA loops trigger transcriptional termination through termination-promoting proteins. Specifically, R-loop dissolving enzymes, such as BRCA1, DHX9, and SETX, come together to resolve the R-loop [118,203]. Once resolved, the R-loop releases nascent RNA for degradation by XRN2, leading to transcriptional termination (Figure 1B) [207].

More genomic experiments are needed to clarify these mechanisms as well as if other classes of genesis require R-loops for transcription termination. Moreover, R-loops trigger antisense transcription, forming double-stranded RNA that facilitates recruitment of RNA interference factors that reinforces H3K9me2 repressive marks to pause RNA Pol II prior to transcriptional termination (Figure 1C) [204].

#### 4.1.3. R-Loops and DNA Double-Strand Break Repair

R-loops are involved in DSB repair as the formation of R-loops is needed for efficient HR [208,209,210]. Genomic location of repair and R-loop processing are several factors that determine R-loops’ role in DSB repair [2]. However, what is universally accepted is that inducing DSBs promotes the formation of RNA–DNA hybrids [211,212]. These subsequent R-loops then play a critical role in several mechanisms that induce HR in human cells [56,213,214,215]. A major influence R-loops have on DSB repair is to alter resection efficiency; however, R-loops play a conflicting role in the mechanism by which it alters resection efficiency [2]. In fission and budding yeast, R-loop formation prevents resection at DSBs, but R-loop removal is required for efficient RPA binding [216]. However, an alternative role is observed in human cells in which R-loops enhance resection at DSBs [212]. Other mechanisms are observed by which R-loops promote DSB repair. One principal mechanism is the initiation of transcription-associated homologous recombination repair (TA-HR) via R-loop formation and resolution [209]. Inducing DSB favors the formation of R-loops which then recruit RAD52 and BRCA1 [209]. RAD52 is crucial for the resolution of RNA–DNA hybrids, and BRCA1 recruits BRCA2 and PALB2, which contributes to the removal of RNA from the hybrid with the help of RNase H1 [217,218]. Next, nucleotide excision repair XPG is recruited and incises the R-loops for the resolution of RNA–DNA hybrids, subsequently promoting TA-HR (Figure 1D) [209]. Another mechanism includes the recruitment of SETX to DSBs, which regulates RAD51 foci formation promoting DSB repair (Figure 1D) [63]. Though R-loops promote DSB repair, they are a potential cause for DNA damage. R-loops can cause faulty repair and block DNA repair proteins from binding to DSBs, leading to potential threats and genome instability.

### 4.2. R-Loops as Threats to Genome Stability

Although R-loops play an important regulated role in transcription activation and termination, unscheduled R-loops are a major source of genome instability through replication fork stalling and the exposure of ssDNA. In R-loop-accumulating cells, genome instability and replication impairment are observed via multiple mechanisms. One such mechanism involves exposed ssDNA. R-loops promote genome instability via exposed ssDNA because of RNA–DNA hybrids formation. These exposed ssDNA are susceptible to nucleases and genotoxins, which results in transcription-associated mutagenesis (TAM) [168]. Several recent studies, however, have proposed another mechanism: R-loop-mediated genome instability, which is caused by the impairment of replication fork progression [38]. Evidence for this mechanism is proven by the recruitment of replication proteins, such as BRCA1 [218], BRCA2 [141], FANCA [135], and FANCD2 [135,136,137], as they are required to resolve R-loops to prevent transcription conflicts.

**Figure 2:**
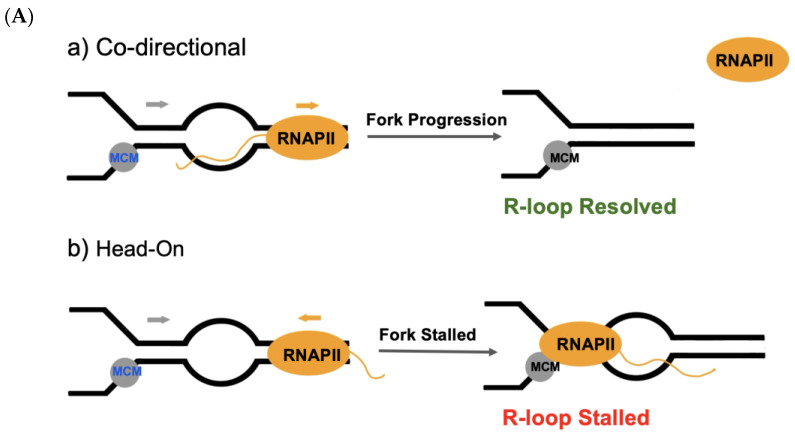

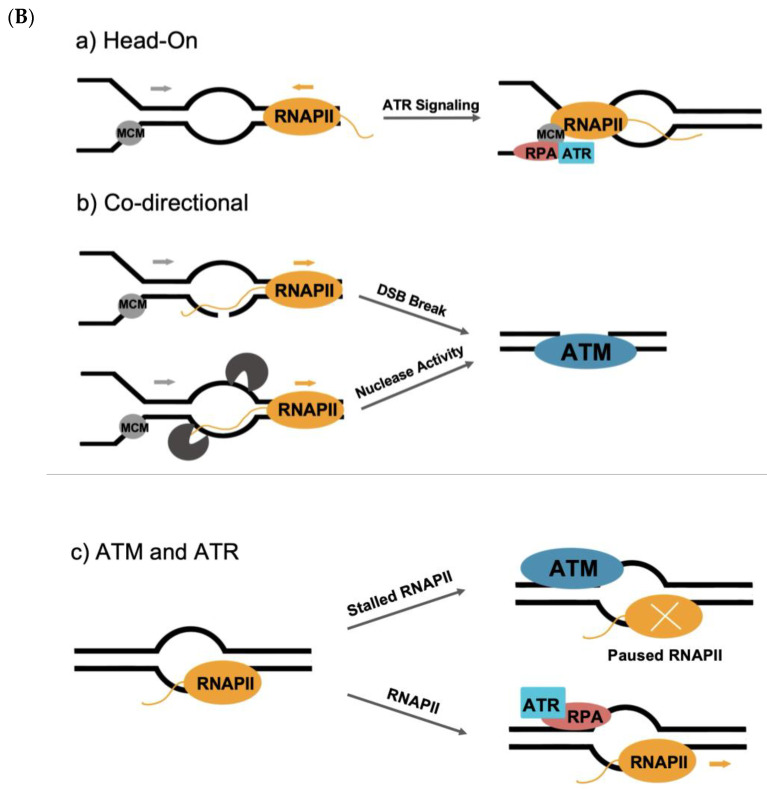
R-loops in genome instability. (**A**) Mechanisms by which HO and CD collisions resolve/stall R-loops. (a) CD collisions resolve and prevent R-loop formation as RNA Pol II moves in the same direction as the DNA strand. (b) HO collision stalls R-loops as RNA Pol II moves in the opposite direction of the DNA strand thus promoting R-loop formation. (**B**) Mechanisms by which ATR and ATM signaling occurs. (a) Head-on collisions promote ATR signaling as RPA binds to ssDNA recruiting ATR. (b) Co-direction promotes ATM signaling via DSBs and nuclease activity. (c) ATM and ATR signaling is also promoted without the presence of replication forks. Stalled RNA Pol II recruits ATM whereas regular RNA Pol II recruits RPA and ATR. Gray arrows: direction of replication. Orange arrows: direction of transcription.

Moreover, in mammalian cells, DNA breaks are predominantly found in the S phase, suggesting a correlation between R-loops and the stalling of replication forks [38]. A detailed mechanism in which R-loops stall replication forks needs further investigation; however, it could account for this stalling of the replication fork to head-on transcriptional regulation (HO-TR) conflicts [168]. In both yeast and bacteria cells, HO-TR depends on the presence of RNA–DNA hybrids, and the persistent presence of the hybrids has been shown to promote replication fork stalling when they are close to HO-TR replication forks [45,219]. Conversely, co-directional transcriptional regulation (CD-TR) conflicts have been shown to maintain genome stability in the presence of R-loops [219,220]. Interestingly, HO collisions promote R-loop formation by primarily stabilizing and preventing the resolution of R-loops (Figure 2A). However, CD collisions have been observed to resolve R-loops and prevent R-loop formation (Figure 2A) [168,220]. These replication forks play a vital role in the regulation of ATR and ATM protein kinases as central regulators of the DNA damage response (DDR). HO collisions are observed to activate ATR-Chk1 where R-loop levels have increased, whereas CD collisions activate ATM-Chk2 where R-loop levels have decreased [35]. The mechanism by which R-loops activate ATM and ATR is not exactly known, but some details are outlined. Co-directional ATM activation may occur when R-loops are converted to DSBs via a nick in the displaced ssDNA or by nuclease activity on the R-loop (Figure 2B) [2]. ATR may be activated at stalled replication forks via HO collisions as RPA is recruited to the ssDNA at the replication fork, which activates ATR (Figure 2B) [2]. R-loops also activate ATM and ATR in the absence of replication forks formed from DSBs. In vivo models of human cells show that in transcription-blocking lesions where RNA polymerase is paused, R-loops promote ATM signaling (Figure 2B) [176]. In addition, R-loops activate ATR through RPA colocalizing with R-loops on ssDNA (Figure 2B) [175]. Interestingly, ATR activation promotes the recruitment of R-loops’ resolution pathways, including SETX to transcription replication sites [171]. Though it may seem as if R-loops are beneficial towards genome stability, once unregulated, R-loops can be detrimental to genome integrity. In human and yeast cells, HO collisions have been shown to be particularly detrimental to genome stability [35,216].

## 5. R-Loops and Cancer

R-loops have been implicated in various cancers by causing DNA damage, genomic instability, and alterations in gene expression. The abnormal accumulation of R-loops has been observed in various cancer types, including hematological malignancies, breast cancer, and other solid tumors [19,30,125,221]. This dysregulation of R-loop formation can arise from multiple factors, including elevated transcription rates, altered chromatin structure, and deficiencies in RNA processing factors [222,223]. Additionally, in the context of cancer, R-loops can interact with both repression factors and oncogenes, further contributing to the complex interplay that drives tumorigenesis [5]. Given R-loops role in driving tumorigenesis, R-loops have strong prognostic potential [224], although currently there is no established medical procedure in utilizing R-loops as a prognostic factor in cancer patients. In this review, we discuss the multifaceted interactions between R-loops and the association with both repression factors and oncogenes.

### 5.1. The Impact of Tumor Suppressor Genes on R-Loops

R-loops exhibit a multifaceted interplay with tumor suppressor genes (TSGs). In controlled conditions, R-loops are involved in modulating gene expression, including the expression of TSGs. However, if R-loops accumulate uncontrolled, they can lead to genomic instability by inducing DNA damage, which may subsequently cause the impairment or silencing of TSGs. This loss of function can disable the standard regulatory mechanisms on cellular growth and division, thereby contributing to the potential onset of cancer [19,160]. The connection between BRCA2 deficiency and the regulation of R-loop levels and associated instability was initially documented in 2014 [141]. Subsequently, numerous investigations have established links between R-loop management and various proteins, including P53, BRCA1, and BRCA2, which are components of the Fanconi anemia pathway, BLM and WRN helicases, and the Mre11–Rad50–Nbs1 complex [121,135,136,137,225,226]. The primary emphasis of these studies has focused on the traditional roles these repair proteins play in responding to halted replication forks, aiding in their stabilization and efficient resumption.

The p53 tumor suppressor, known for its roles in transcription, apoptosis, and cell-cycle arrest, is critical in stabilizing DNA replication forks and preventing genomic instability, independent of its transcriptional activity. When p53 is defective or depleted, it hijacks stalled replication forks by mutagenic pathways like RAD52 and POL θ. These mutagenic pathways have been shown to contribute to genomic instability, observed in the mutation patterns of p53-deficient breast cancer patients, suggesting that P53 could play a role in R-loop suppression [227]. Moreover, one study reveals that R-loops are formed due to the lack of various RNA processing factors or due to the inhibition of topoisomerase I and are actively converted into DSBs through the action of the tumor suppressor XPF and XPG. Thus, these observations disclose an unanticipated and potentially harmful function of transcription-coupled nucleotide excision repair (TC-NER) factors in promoting R-loops’ resolution and facilitating R-loop-induced DNA damage and genomic instability [109].

The Fanconi anemia (FA) pathway, which comprises at least 22 genes and orchestrates various processes, plays a crucial role in DNA interstrand crosslinks (ICLs) during replication, where it initiates fork remodeling, strand cleavage, translesion synthesis, and homologous recombination [134]. Research has delved into the non-traditional roles of the Fanconi anemia (FA) pathway in resolving R-loops. For instance, FANCM has been demonstrated to have the capability to eradicate R-loop structures in a controlled environment and has the potential to directly dissolve them in cells if directed to an R-loop site [136]. Research has delved into the non-traditional roles of the Fanconi anemia (FA) pathway in resolving R-loops. For instance, FANCM has been demonstrated to have the capability to eradicate R-loop structures in a controlled environment and has the potential to directly dissolve them in cells if directed to an R-loop site [137].

Numerous studies suggest that BRCA1 and BRCA2 regulate R-loops, emphasizing these proteins’ roles in controlling gene expression. It is demonstrated that BRCA1 is drawn to R-loops formed at certain transcription termination regions and is crucial for bringing in SETX, its natural binding partner. When the BRCA1/SETX complex is disrupted, it damages R-loop-induced DNA. Additionally, BRCA1 has been pinpointed to exhibit a strong affinity for R-loop-enriched termination zones in active transcription genes. Notably, its absence in breast carcinomas is linked to distinct mutations proximate to these regions, thereby accentuating the pivotal role of the BRCA1/SETX consortium in mending DNA impairments at transcriptional stalling sites instigated by R-loops [109]. As for BRCA2, it is a human tumor suppressor that has a role in aiding the transition from promoter-proximal pausing to efficient transcription elongation by fostering the recruitment of PAF1 to RNA Pol II, in which this interaction is crucial for thwarting the accumulation of harmful RNA–DNA hybrids known as R-loops. In circumstances where BRCA2 is incapacitated, either by depletion or malignancy-associated mutations, there is a conspicuous accumulation of RNA Pol II. This cascade culminates in the escalation of R-loops at promoter-proximal pausing locales within genes engrossed in active transcription, paving the way for DNA detriment—a phenomenon intimately intertwined with diminished PAF1 conscription and stymied RNA synthesis [139].

### 5.2. The Influence of Oncogenes on R-Loops

As oncogenes propel cells toward rapid division, there is also an increase in transcription activity, which raises the probability of R-loops’ formation. The activation of oncogenes by R-loops often triggers alterations in transcriptional activities, which promotes cancer hallmarks [41]. These changes in transcription can lead to conflicts between transcription and DNA replication, known as TRCs. The timing of DNA replication, in conjunction with the forced entry into the cell cycle instigated by oncogenes, might clash with the altered transcriptional profile. This clash can foster the formation of R-loops at the sites of TRCs, where these R-loops can contribute to replication stress, a common feature in cancer. It can further promote genomic instability and disease progression [228].

Alterations or imbalances in oncogene activity have been connected to DNA damage associated with R-loops through various mechanisms. For instance, when the oncogene HRASV12 is overexpressed, there is an increase in RNA synthesis due to the elevated expression of the transcription factor TATA-box-binding protein (TBP). This uptick in transcription, combined with the accumulation of R-loops, results in the slowing down of replication forks and DNA damage, thereby establishing a connection between enhanced transcriptional activity and genomic instability in cancer [41]. Transcriptional activation during oncogenesis likely gives rise to R-loops and TRCs. Oncogenic fusion transcription factors linked to sarcoma, like EWS-FLI and SS18-SSX, have been demonstrated to promote R-loops’ formation, contributing to DNA replication stress [229,230]. When the oncogene Cyclin E is overexpressed in cells in the S phase of cell division, excessive replication origin activation leads to hindered replication fork progression and DNA damage, activating RAD51-mediated recombination. The study suggests that the replication stress induced by Cyclin E is due to deregulated replication initiation and heightened conflicts between replication and transcription, culminating in impeded replication fork progression and DNA damage that either activates tumor-suppressing mechanisms or fosters cancer-promoting mutations [231]. The initiation or reconfiguration of transcription is adequate to induce DNA damage associated with R-loops. The standard increase in transcription caused by estrogen stimulation in MCF7 cells, which is not observed in MCF10A cells without estrogen receptors, results in unscheduled R-loops’ formation and subsequent DNA damage [42]. Mutations in splicing factors U2AF1 and SRSF2, which are associated with myelodysplastic syndrome (MDS), have been linked to R-loops and have functional incapacitation variants in SF3B1 in a zebrafish model [189,190,232]. It is hypothesized that these effects do not arise from an increase in overall transcription but rather from changes in splicing patterns that either directly facilitate R-loop formation in proximity to irregular splicing events or indirectly encourage R-loop formation through alterations in gene expression [233]. In addition, Mdm2 and PRC1, mainly known as component RNF2/Ring1B, play a vital role in ensuring the smooth progression of DNA replication forks, where ubiquitination and deubiquitination of H2A are crucial. It also suggests that Mdm2, through chromatin modification, helps prevent the formation of R-loops’ structures comprising RNA–DNA hybrids, which can impede DNA replication [179].

These findings indicate that widespread disruptions in transcription, RNA processing, or DNA replication brought about by oncogenes can lead to increased R-loop formation and consequent DNA damage. In most instances, overexpressing RNase H1 mitigates DNA damage or replication stress symptoms, suggesting that R-loops might contribute to TRCs or need to be resolved for efficient TRC mitigation [41,229,230].

## 6. Technologies for Mapping of Genomic R-Loops

Given R-loops’ evidentiary role in genomic regulation and pathologic conditions, genome-wide mapping techniques are critical to profiling R-loop formation. Mapping techniques that detect changes in R-loop formation and characterize R-loop features associated with pathological conditions are crucial to the understanding of R-loop mechanistic properties and the development of potential therapies associated with R-loop-promoted diseases, such as cancer. Indeed, several techniques have been developed to detect genome-wide R-loops. These techniques include the use of S9.6 monoclonal antibodies and catalytically inactive RNase H’s that predominantly recognize RNA–DNA hybrids [2,10,234,235]. Here, we briefly review these genomic mapping techniques, discussing their unique advantages and disadvantages, as well as new emerging technologies developed to profile genome-wide R-loop formation.

### 6.1. The S9.6 Monoclonal Antibody-Based Approach

Utilizing S9.6 and next-generation sequencing, DNA–RNA immunoprecipitation sequencing (DRIP-seq) is the most widely adopted approach to map genomic R-loops [10]. DRIP-seq is a simple technique by which nucleic acids are extracted and sheared via restriction enzyme [2]. R-loops are then immunoprecipitated by the S9.6 antibody. Targeted, precise enrichment of R-loops is estimated using quantitative polymerase chain reaction (qPCR) or reverse transcription qPCR to confirm that R-loops contain RNase H-sensitive RNA molecules [234]. Though a widely adopted technique, DRIP-seq suffers from limited resolution and unspecified strand specificity [236].

To counteract limited resolution and strand insensitivity, variations of DRIP-seq have been studied and developed. RDIP-seq is an optimized variation of DRIP-seq that utilizes RNase I on extracted nucleic acids and sonification to minimize bias in nucleic acid fragmentation [237]. Though RDIP-seq derives strand-specific signaling and optimized resolution [2], it requires the use of sonification that has been shown to disrupt the R-loop structure, impairing accurate R-loop mapping [238]. Sonification is also used in S1-DRIP-seq, which employs S1 nucleases to digest R-loops displaced ssDNA, thereby stabilizing R-loops through the sonification process, allowing for accurate R-loop mapping. However, S1-DRIP-seq is not strand-specific [238]. To counteract strand insensitivity, DRIPc-seq was developed to sequence RNA from the RNA–DNA hybrid in a strand-specific manner to near-nucleotide resolution. DRIPc-seq utilizes the DRIP-seq procedure with the addition of cDNA conversion after immunoprecipitation of R-loops [239]. Additionally, the ssDNA ligation-based library construction after R-loop immunoprecipitation combined with next-generation sequencing (ssDRIP-seq) was developed to map R-loops with fewer steps for library construction and strand specificity. ssDRIP-seq sequences the template strand hybridized to the R-loop RNA [240]. Though a simpler method to sequence R-loops compared to DRIP-seq, ssDRIP-seq suffers from low resolution of R-loop mapping [2]. Lastly, bisDRIP-seq utilizes the combination of bisulfite foot printing of the R-loops ssDNA and S9.6 to immunoprecipitate the bisulfite-modified R-loops, theoretically mapping R-loops at near-nucleotide resolution [198]. Though an efficient method to map R-loops, bisDRIP-seq typically underestimates the size of R-loops and is a difficult method to implement and analyze [234].

Though an efficient technique to map genome-wide R-loops, the S9.6 approach relies on S9.6 having a specified, unbiased affinity on R-loops. However, S9.6 has been shown to have a binding affinity to RNA–RNA hybrids, albeit at a weaker affinity, in fission yeast [241]. Thus, S9.6 may not have a specified, biased affinity to RNA–DNA hybrids, overestimating R-loop formation. Additionally, S9.6 has an epitope length of 6bp; thus, S9.6 may not recognize R-loops smaller than 6bp, underestimating R-loop formation [242].

### 6.2. Inactive RNase H-Based Approach

As discussed in detail in Section 3.1, RNase H1 is a ribonuclease that dismantles RNA strands in RNA–DNA hybrids, thus exhibiting high binding affinity to R-loops. Due to its ability to recognize R-loop structures, RNase H1 has been used to map genomic R-loops. In particular, a mutant RNase H1 (dRNASEH1) is developed to bind to RNA moieties in RNA–DNA hybrids but inhibits its catalytic activity in degrading R-loops [10,243,244]. DRIVE-seq utilizes dRNASEH1 to locate and bind to R-loops. An affinity pull-down assay is conducted with next-generation sequencing to map genomic R-loops in vitro [2,10]. However, DRIVE-seq is less sensitive than DRIP-seq, computing 1224 peaks compared to DRIP-seq, which computes 20,862 peaks [10]. To improve sensitivity, R-ChIP was developed, which employs exogenous catalytically inactive RNASEH1, chromatin immunoprecipitation (ChIP), and a strand-specific library for sequencing to map genomic R-loops in vivo. Specifically, in R-ChIP, dRNASEH1 is transfected in cells to bind to RNA–DNA hybrids, followed by ChIP of the tagged dRNASEH1 and sequencing for R-loop mapping. R-ChIP sequences R-loops with high specificity and resolution [244]. However, like DRIVE-seq, R-ChIP underestimates R-loop formation and provides an incomplete R-loop map. As discussed in Section 3, many proteins are involved in the suppression and resolution of R-loops that have similar functions to RNase H’s. DRIVE-seq and R-ChIP does not utilize these various proteins and only sequences R-loops recognized by RNase H1, possibly providing an explanation for DRIVE-seq and R-ChIP underestimating R-loop formation in a genomic map [234,244]. To counteract these downfalls, complementary approaches of various R-loop mapping techniques must be employed to provide an accurate, complete genomic R-loop map. Moreover, future efforts in developing R-loop mapping techniques may help address these limitations. However, to date there is no “gold standard” approach to mapping genomic R-loops.

## 7. Conclusions

It has become clear that R-loops as distinct and dynamic RNA–DNA–protein assemblies are not just by-products of transcription but play significant roles in cellular processes. R-loops have imperative impacts on the regulation of gene expression, DNA replication initiation, transcription termination, DNA repair, and histones and chromatin modification states on an epigenetic level, all of which are important for normal cell physiology. However, unregulated R-loop accumulation has been demonstrated to contribute to pathological consequences, including genomic instability through replication stress, DNA breaks, transcription-associated mutations, altered gene expression, and epigenetic alterations. Such adverse impacts contribute to the development of numerous pathological health conditions, including cancer, where abnormal R-loop accumulation may drive oncogenesis.

Studying the biochemistry of R-loops could help elucidate the mechanisms driving carcinogenesis for the identification of potential oncogenic therapeutic targets. An intriguing prospect is the design of therapeutics poised to amplify R-loop genesis or stymie their resolution, especially within cancer cells that bear compromised DNA repair faculties. Such targeted therapies could selectively impact cancer cells, offering more specialized and effective treatment options. Thus, future research and development could radically transform cancer treatment and improve patient outcomes. Despite significant strides in our comprehension of R-loops, there remain numerous challenges. The molecular mechanisms of R-loop formation and their regulation still need to be fully understood, requiring broadened research efforts to pinpoint and authenticate potential therapeutic targets within the R-loop metabolism pathway. Furthermore, forging small molecular entities that adeptly and selectively regulate in vivo R-loop concentrations presents a formidable scientific challenge warranting relentless inquiry.

In summation, while the precise relationship between R-loops, genome instability, and cancer is intricate and multifaceted, investigating this relationship provides an increasingly nuanced understanding of cancer biology. As we continue to evolve our understanding of R-loops’ biochemical processes, it brings hope for the design of more specialized and efficacious cancer therapies in the future.
